# Sphingomyelin Depletion Inhibits CXCR4 Dynamics and CXCL12-Mediated Directed Cell Migration in Human T Cells

**DOI:** 10.3389/fimmu.2022.925559

**Published:** 2022-07-12

**Authors:** Sofía R. Gardeta, Eva M. García-Cuesta, Gianluca D’Agostino, Blanca Soler Palacios, Adriana Quijada-Freire, Pilar Lucas, Jorge Bernardino de la Serna, Carolina Gonzalez-Riano, Coral Barbas, José Miguel Rodríguez-Frade, Mario Mellado

**Affiliations:** ^1^ Chemokine Signaling Group, Department of Immunology and Oncology, National Center for Biotechnology/Consejo Superior de Investigaciones Científicas, Madrid, Spain; ^2^ National Heart and Lung Institute, Imperial College London, London, United Kingdom; ^3^ Central Laser Facility, Rutherford Appleton Laboratory, Medical Research Council-Research Complex at Harwell, Science and Technology Facilities Council, Harwell, United Kingdom; ^4^ National Institute for Health and Care Research Imperial Biomedical Research Center, London, United Kingdom; ^5^ Metabolomic and Bioanalysis Center (CEMBIO), Pharmacy Faculty, Centro de Estudios Universitarios Universities, Madrid, Spain

**Keywords:** chemokines, chemokine receptors, sphingolipids, ceramides, cell migration

## Abstract

Sphingolipids, ceramides and cholesterol are integral components of cellular membranes, and they also play important roles in signal transduction by regulating the dynamics of membrane receptors through their effects on membrane fluidity. Here, we combined biochemical and functional assays with single-particle tracking analysis of diffusion in the plasma membrane to demonstrate that the local lipid environment regulates CXCR4 organization and function and modulates chemokine-triggered directed cell migration. Prolonged treatment of T cells with bacterial sphingomyelinase promoted the complete and sustained breakdown of sphingomyelins and the accumulation of the corresponding ceramides, which altered both membrane fluidity and CXCR4 nanoclustering and dynamics. Under these conditions CXCR4 retained some CXCL12-mediated signaling activity but failed to promote efficient directed cell migration. Our data underscore a critical role for the local lipid composition at the cell membrane in regulating the lateral mobility of chemokine receptors, and their ability to dynamically increase receptor density at the leading edge to promote efficient cell migration.

## Introduction

Directed cell migration is fundamental for the spatio-temporal positioning of leukocytes and ensures the organization and function of the immune system. It involves the complex integration of several extracellular stimuli coordinated by many intracellular signaling events including the synchronized action of the actin cytoskeleton, which enables cell and receptor polarization (1, 2), and allows the spatial perception of chemoattractant gradients. Lamellipodia and filopodia at the leading edge of migrating cells sense directional information (3), which is translated into the dynamic assembly of actin filaments pushing the leading edge forward until membrane tension-generated forces lead to their retraction (4–6).

Chemokines are critical for immune reactions, as they direct leukocyte recruitment under both homeostatic and inflammatory conditions. They act by binding to members of the G protein-coupled receptor (GPCR) superfamily localized at the leading edge of migrating cells. Accumulating data indicate that chemokine receptors can exist not only as monomeric entities, but they can also associate into both homo and heterodimers, and even into higher-order oligomers, which ensures ligand-mediated function (7–10). Using high spatial resolution analysis, we previously reported that CXC motif chemokine receptor type 4 (CXCR4) forms basal nanoclusters in resting T cells, the extent, dynamics and signaling strength of which are modulated by the orchestrated action of the actin cytoskeleton and the presence of its ligand CXCL12 (8). We also found that a mutant CXCR4 that is expressed at the cell membrane and dimerizes but does not form large nanoclusters, and it is unable to promote directed cell migration (8). Like all other integral membrane proteins, chemokine receptors are embedded in a compartmentalized lipid bilayer environment, which influences their dynamics (11). Indeed, the association between CXCR4 and lipid-enriched membrane microdomains has been reported to be functionally important (12, 13). Similarly, cholesterol is known to modulate chemokine receptor function (14).

As essential components of cellular membranes, sphingolipids participate in myriad cellular processes requiring membrane integrity and dynamics (15). Their composition and high abundance within the plasma membrane and their dynamic changes have a substantial impact on membrane biophysics and, by extension, on the dynamics of embedded proteins (16, 17). Sphingolipids and cholesterol regulate membrane fluidity, which is important for membrane deformability during inward/outward vesiculation, endo/exocytosis and cell polarization (16, 17). They also play key roles in the compartmentalization of membrane proteins and associated signaling components within membrane domains formed in steady-state or in response to stimulation (18, 19). Ceramide-enriched membrane domains formed by sphingomyelin breakdown in the external part of the cell membrane serve as platforms for signal relay and initiation, which often directly involves the regulation of membrane proximal cytoskeletal dynamics (20–23). Analogously, dynamic alterations of local membrane domains are important for the organization and maintenance of receptor-mediated processes such as cell adhesion and migration, signal perception and cytoskeletal coupling (24).

Ceramide and neutral sphingomyelinase 2 (NSM2), an enzyme that metabolizes sphingomyelins, accumulate within the lamella in co-stimulated T cells (25), and CD4^+^ cells lacking NSM2, fail to polarize and redistribute CXCR4, and exhibit impaired directional migration in response to CXCL12 (26). Likewise, pharmacological NSM2 inhibition blocks directionality of human polymorphonuclear neutrophils in response to the bacterial peptide FMLP, but not their overall motility (27). Measles virus infection is known to overactivate NSM2, which causes cytoskeletal paralysis, whereas inhibiting NSM2 activity appears to substantially enhance T cell responses and, at the cytoskeletal level, increases adhesion and spreading (28). These observations clearly support a major role for sphingomyelin metabolism in the local regulation of the actin cytoskeleton under physiologic conditions (25) and in the organization of chemokine receptors in membrane domains.

Here, using CXCR4 and CXCL12 as a model, and using single-particle tracking (SPT) in total internal reflection fluorescence (TIRF) mode, we investigated how extended sphingomyelin depletion alters CXCR4 nanoclustering and dynamics at the T cell membrane. Our results, supported by lipidomics, indicate that prolonged exogenous treatment of Jurkat cells and T cell blasts with bacterial sphingomyelinase (bSMase) promotes the complete and sustained breakdown of sphingomyelins to generate the corresponding ceramides in cell membranes. Under these conditions, membrane fluidity was increased, and this was accompanied by altered CXCR4 dynamics and defects in some CXCL12-mediated functions. Fluorescence resonance energy transfer (FRET) studies confirmed that bSMase treatment influences the conformation of CXCR4 at the cell membrane and SPT/TIRF analysis indicated that CXCL12 failed to trigger CXCR4 nanoclustering. As a consequence, cells were unable to properly sense chemoattractant gradients.

## Materials and Methods

### Cells and Reagents

HEK-293T cells were obtained from the ATCC (CRL-11268) and human Jurkat leukemia CD4^+^ cells were kindly donated by Dr. J. Alcamí (Centro Nacional de Microbiología, Instituto de Salud Carlos III, Madrid, Spain). When needed, Jurkat cells lacking endogenous CXCR4 expression (Jurkat^-/-^) (29) were transiently transfected with CXCR4-AcGFP (20 μg; JK^-/-^X4) using a BioRad electroporator (20 × 10^6^ cells/400 μL RPMI 1640 with 10% fetal calf serum. 280V, 975 μF) and analyzed 24 hours later. Human peripheral blood mononuclear cells were isolated from buffy coats by centrifugation through Ficoll-Paque PLUS density gradients (GE Healthcare, Wakuesha, WI) at 760 × *g* for 30 minutes at room temperature (RT). They were then *in vitro* activated for 1 week with 20 U/mL of IL-2 (Teceleukin; Roche, Nutley, NJ) and 5 μg/mL phytohemagglutinin PHA (Roche) to generate T cell blasts (30).

The following antibodies were used: non-conjugated monoclonal anti-human CXCR4 (clone 44717), anti-CXCR4 (12G5) and anti-CD4 (OKT4) conjugated to PE and FITC accordingly, all from R&D Systems (Minneapolis, MN); anti-phospho-ERK1,2 (#9191), anti-ERK (#9102), anti-pAkt (#4060) and anti-Akt (#9272) from Cell Signaling Technology (Danvers, MA); phalloidin-TRITC (#P195) from Sigma-Merck (Darmstadt, Germany); and anti-GFP (JL-8) from Clontech Laboratories (Palo Alto, CA). Human CXCL12 and CCL19 were purchased from PeproTech (Rocky Hill, NJ). Human CXCR4 was cloned into pECFP-N1, pEYFP-N1 and pAcGFPm-N1 vectors (Clontech Laboratories), as described (8). Bacterial sphingomyelinase (bSMase, Staphylococcus aureus SMase, #S9396) was obtained from Sigma-Aldrich (Madrid, Spain) and the Di-4-ANEPPDHQ probe was from Invitrogen (Carlsbad, CA).

### Lipidomics Analysis by UHPLC-ESI-QTOF MS

For lipid extraction from Jurkat and T cell blasts, cell pellets (1 x 10^7^ cells/pellet) were mixed with 200 μL of cold (-20°C) methanol:water (1:1, v/v) and sonicated with an ultrasonic homogenizer (UP200S, Hielscher Ultrasound Technology, HIELSCHER GmbH, Chamerau, Germany) for 16 bursts (0.5 second pulse) at 80% amplitude. Homogenates (100 μL) were mixed with 320 μL of cold (-20°C) methanol containing 1.6 ppm of sphinganine (d17:0) as the internal standard for positive-ion electrospray ionization (ESI) and 3.9 ppm of palmitic acid-d31 as the internal standard for negative-ion ESI. Samples were then vortex-mixed for 2 minutes, followed by the addition of 80 μL of methyl tert-butyl ether. Subsequently, samples were vortex-mixed (1 hour, RT). After centrifugation (16,000 *× g*, 15°C, 10 minutes), samples were used for ultra-high performance liquid chromatography (UHPLC; Agilent 1290 Infinity II, Agilent Technologies Inc., Santa Clara, CA) coupled with (ESI) quadrupole time-of-flight (QTOF) mass spectrometry (MS) (Agilent 6546): 100 μL of each sample was divided between two UHPLC-MS vials with inserts (50 μL/each) for direct injection into the system for LC-MS analyses in positive and negative ionization modes.

Quality control and blank samples were prepared for each cell population by pooling equal volumes of each sample. Samples were then randomized within each cell population, and quality controls were injected at the beginning, after every five experimental samples, and at the end of the batch. Blank samples were analyzed at the beginning and at the end of the analytical sequence. Finally, 10 iterative-MS/MS runs/group were performed for both ion modes at the end of the analytical run at 20 and 40 eV.

Raw LC-MS/MS data were reprocessed with Lipid Annotator software (Agilent Technologies). The MS/MS spectra of the 20 sphingomyelin and 20 ceramide candidates obtained were manually inspected using Agilent MassHunter Qualitative (v10.0), comparing the retention time and MS/MS fragmentation to the available spectral data across several databases included in the CEU Mass Mediator tool (CMM) (http://ceumass.eps.uspceu.es/mediator/) (31). MassHunter Profinder software (B.10.0.2, Agilent Technologies) was used to clean the raw LC-MS data from background noise and unrelated ions for feature building for the sphingomyelin and ceramide lipid species detected, and for feature alignment across the study samples (32). Data normalization was performed before statistical analysis. The raw data matrices were normalized according to the intensity of the corresponding internal standard to correct the unwanted variance related to sample preparation and the analytical run. Then, the coefficient of variation of the detected sphingomyelin and ceramide lipid species was evaluated in the quality controls, with all displaying a coefficient of variation <10%. The original and crude data from these experiments has been deposited at Metabolomics Workbench platform (https://www.metabolomicsworkbench.org) (access no. ST002150).

### Western Blotting

Cells (3 × 10^6^) were activated with CXCL12 (50 nM) at the indicated time points and then lysed in RIPA detergent buffer supplemented with 1 mM PMSF, 10 μg/mL aprotinin, 10 μg/mL leupeptin and 10 μM sodium orthovanadate for 30 minutes at 4°C. Extracts were analyzed by western blotting using specific antibodies. Densitometric evaluation of western blot experiments was performed using ImageJ software.

### Flow Cytometry Studies

Cells were incubated with specific antibodies (30 minutes, 4°C) and mean fluorescence intensity was determined on a FC500 flow cytometer (Beckman Coulter Inc., Brea, CA). Receptor internalization was determined by flow cytometry after activation with CXCL12 (50 nM) at the indicated time points. Results are expressed as a percentage of the mean fluorescence intensity of treated cells relative to that of unstimulated cells.

### Annexin-V 7AAD/Propidium Iodide Staining

Cultured cells were collected from microplates and washed twice in PBS buffer before staining with Annexin-V PE and 7AAD (Immunostep, Spain) following the manufacturer’s instructions or propidium iodide (3 μM, 30 minutes, RT) for analysis by flow cytometry (FC500, Beckman Coulter). Results are expressed as % of stained cells.

### Transwell Migration Assay

Cells (3 × 10^5^ in 0.1 mL of RPMI containing 10 mM HEPES and 0.1% BSA) were placed in the upper wells of uncoated 24-well transmigration chambers (3-μm pore or 5-μm pore; Transwell, Costar, Corning, NY). CXCL12 (12.5 nM) in 0.6 mL of the same medium was added to the lower well. Plates were incubated for 120 minutes (37°C, 5% CO_2_) and cells that migrated to the lower chamber were counted by flow cytometry (FC500, Beckman Coulter), corrected for variations in input concentrations, and expressed as the mean (standard deviation, SD) percentage of cell migration. When required, cells were pretreated with bSMase (0.5 U/mL, 60 minutes, 37°C) and then maintained during cell migration.

### Raster Image Correlation Spectroscopy Analysis

Cells were seeded (30 minutes, 37°C) on Ibidi μ-well-chambers (Martinsried, Germany) coated with fibronectin (20 μg/mL, 30 minutes, 37°C, Sigma) or poly-L-lysine (20 μg/mL, 30 minutes, 37°C, Sigma). Adhered cells were untreated or treated with bSMase (0.5 U/mL, 60 minutes, 37°C) prior to imaging. The Di-4-ANEPPDHQ probe was added (5 μM) to wells just before imaging. Cells were imaged in phenol-free medium supplemented with 10 mM HEPES and 0.1% BSA, at 37°C and 5% CO_2_.

Time-lapse images of cells were acquired on a Leica TCS SP5 inverted confocal microscope (Leica Microsystems, Wetzlar, Germany) fitted with an HCX PL APO 63×/1.2 NA water immersion objective. Di-4-ANEPPDHQ was excited using an argon white light laser at 488 nm. Emission signals were collected with two photomultiplier tubes (500–580, 620–750) and the pinhole was set to one Airy unit. Optimized acquisition was performed to retrieve membrane diffusion values as described (33, 34). Images of 256 × 256 pixels at 8-bit depth were collected using 80.4 nm pixel size and 4 μs dwell time, for 200 consecutive frames.

Raster image correlation spectroscopy (RICS) analysis was performed with “SimFCS 4” software (Global Software, G-SOFT Inc., Champaign, IL), as described (35). RICS analysis was performed in regions of interest of 64 × 64 pixels at 4 random cytoplasmic areas per cell using a moving average (background subtraction) of 10 to discard possible artefacts due to cellular motion and slow-moving particles passing through. The autocorrelation 2D map was then fitted to obtain a surface map that was represented as a 3D projection with the residuals on top. As a general rule, we focused on those regions with intensity fluctuation events in which the intensity changes were following short increasing or decreasing steps, avoiding abrupt intensity decays or increases.

### Total Internal Reflection Fluorescence Analysis

Jurkat^-/-^ cells were transiently transfected with CXCR4 receptor fused to the AcGFP monomeric protein (JK^-/-^X4). Twenty-four hours after transfection, cells expressing low receptor-AcGFP^+^ levels were selected by cell sorting on a BD FACSAria Fusion (BD Biosciences) platform for detection and tracking analysis. CXCR4 expression levels were then evaluated using the Dako Cytomation Qifikit and flow cytometry as described (36). Transfected cells expressing ~8,500–22,000 receptors/cell, which equates to a density of <4.5 particles/μm^2^, were selected for detection and tracking analysis.

Experiments were performed with cells untreated or treated with bSMase (0.5 U/mL, 60 minutes, 37°C) using a TIRF microscope (Leica AM TIRF inverted) equipped with a Hamamatsu Flash 4 digital sCMOS camera (Hamamatsu Photonics Europe GmbH, Herrsching, Germany), a 100× oil-immersion objective (HCX PL APO 100×/1.47 NA) and a 488-nm diode laser. The microscope was equipped with an incubator and temperature control units; experiments were performed at 37°C with 5% CO_2_. Image sequences of individual particles (500 frames) were then acquired at 3.5% laser power with a frame rate of 10 Hz (90 ms/frame). Penetration depth of the evanescent field was 90 nm.

Particles were detected and tracked using described algorithms (U-Track2 (37);) implemented in MATLAB, as described (8). Mean spot intensity (MSI), number of mobile and immobile particles and diffusion coefficients (D_1:4_) were calculated from the analysis of thousands of single trajectories over multiple cells (statistics provided in the respective figure captions), using described routines (38). Receptor number along individual trajectories was determined as described (8). We measured the average fluorescence intensity for the first 20 frames of each trajectory and used the intensity of the monomeric protein CD86-AcGFP as a reference in Jurkat^-/-^ cells transiently transfected with CD86-AcGFP. Distribution of monomeric particles intensities was analyzed by Gaussian fitting, rendering a mean value of 69.33 ± 3.26 a.u. A similar intensity value of the monomer was obtained for CXCR4-AcGFP particles showing a unique photobleaching step. Therefore, this value was used as the monomer reference to estimate the number of CXCR4-AcGFP molecules per particle (**Supplemental Figures 1A, B**).

### Fluorescence Resonance Transfer Analysis by Sensitized Emission

HEK-293T cells transiently transfected at a fixed CXCR4-YFP : CXCR4-CFP ratio (15 μg and 9 μg, respectively) were treated with bSMase (0.5 U/mL, 60 minutes, 37°C) or its solvent (50 mM Tris-HCl; pH 7.5) as a control, and FRET efficiency was evaluated (n = 3, mean ± SEM, ∗∗∗p ≤ 0.001). Emission light was quantified using the Wallac Envision 2104 Multilabel Reader (Perkin Elmer, Foster City, CA) equipped with a high-energy xenon flash lamp (donor: receptor fused to C-CFP, 8-nm bandwidth excitation filter at 405 nm; acceptor: receptor fused to YFP, 10 nm bandwidth excitation filter at 510 nm).

### Directional Cell Migration

Chambers (Ibidi μ−Slide Chemotaxis System; 80326) were first coated with fibronectin (20 μg/mL, 60 minutes, 37°C). Subsequently, untreated or bSMase-treated (0.5 U/mL, 60 minutes, 37°C) cells were diluted to 10 × 10^6^ cell/mL in RPMI medium containing 1% BSA and 20 mM HEPES (chemotaxis medium), seeded into the channel of the chemotaxis slide, and cultured (60 minutes, 37°C). The reservoirs were then filled with chemotaxis medium and 50 nM CXCL12 was added to the right reservoir. Phase-contrast images were recorded over 20 hours with a time lapse of 2 minutes using a Microfluor inverted microscope with a 10× objective (Leica) and equipped with an incubation system set to 5% CO_2_ and 37°C. Single-cell tracking was evaluated by selecting the center of mass in each frame using the manual tracking plug-in tool in ImageJ (NIH, Bethesda, MD). Spider plots, representing the trajectories of the tracked cells, forward migration index (FMI), and straightness values were obtained using the chemotaxis and migration plug-in tool (https://ibidi.com/chemotaxis-analysis/171-chemotaxis-and-migration-tool.html, Ibidi).

### Immunofluorescence Analyses

T cell blasts, treated or not with bSMase (0.5 U/mL, 3 hours, 37°C, 5% CO_2_), were plated on fibronectin (20 μg/mL, Sigma)-coated glass slides and were then stimulated or not with 100 nM CXCL12 or CCL19 (5 minutes at 37°C) and fixed with 4% paraformaldehyde (10 min, RT). Preparations were blocked with PBS containing 150 mM NaCl, 0.1% goat serum and 1% BSA (60 minutes, RT) before staining with anti-human ICAM-3 or anti-CXCR4 (44717) plus AlexaFluor 488 goat anti-mouse IgG (30 minutes, RT, Thermo Fisher Scientific). Cells were permeabilized with 0.25% saponin (10 minutes, RT) and stained with phalloidin-TRITC (Sigma-Merck; 30 minutes, RT). Preparations were analyzed using a ZEISS confocal multispectral microscope.

### Statistical Analysis

Data were analyzed using GraphPad PRISM (ns = not significant p>0.05; *p ≤ 0.05; ** p ≤ 0.01; *** p ≤ 0.001; **** p ≤ 0.0001). Data from experiments of cell migration, directional cell migration assays, RICS, and MSI, were analyzed using one-way ANOVA followed by Tukey’s multiple comparison test. A two-tailed Mann-Whitney non-parametric test was used to analyze the diffusion coefficient (D1-4) of single particles and FRET efficiency. We used contingency tables to compare two or more groups of categorical variables, such as the percentages of mobile or immobile particles, and these were compared using the Chi-square test with a two-tailed p-value. For lipidomic analysis, statistics were analyzed using Matlab (R2018a, MathWorks), by the Mann-Whitney U test (p ≤ 0.05) after normality testing with the Shapiro-Wilk test. The false discovery rate at the level α = 0.05 was inspected by the Benjamini–Hochberg correction test.

## Results

### Prolonged Sphingomyelinase Treatment Diminishes CXCR4 Dependent T Cell Migration

**Figure 1:**
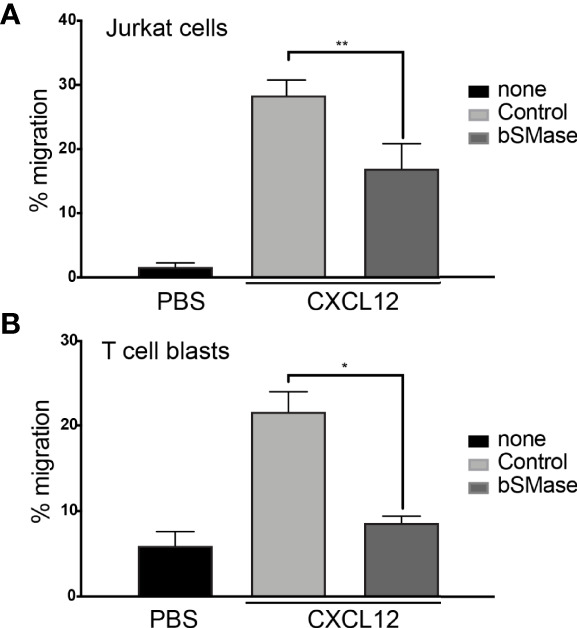
bSMase treatment alters CXCL12-mediated cell chemotaxis. **(A)** Migration of untreated and bSMase-treated Jurkat cells in response to CXCL12 12.5nM. Data are shown as the mean percentage (plus SD) of input cells that migrate (n = 4; **p ≤ 0.01). **(B)** Migration of untreated and bSMase-treated T cell blasts in response to CXCL12. Data are shown as the mean percentage (plus SD) of input cells that migrate (n = 3; *p ≤ 0.05).

**Figure 2:**
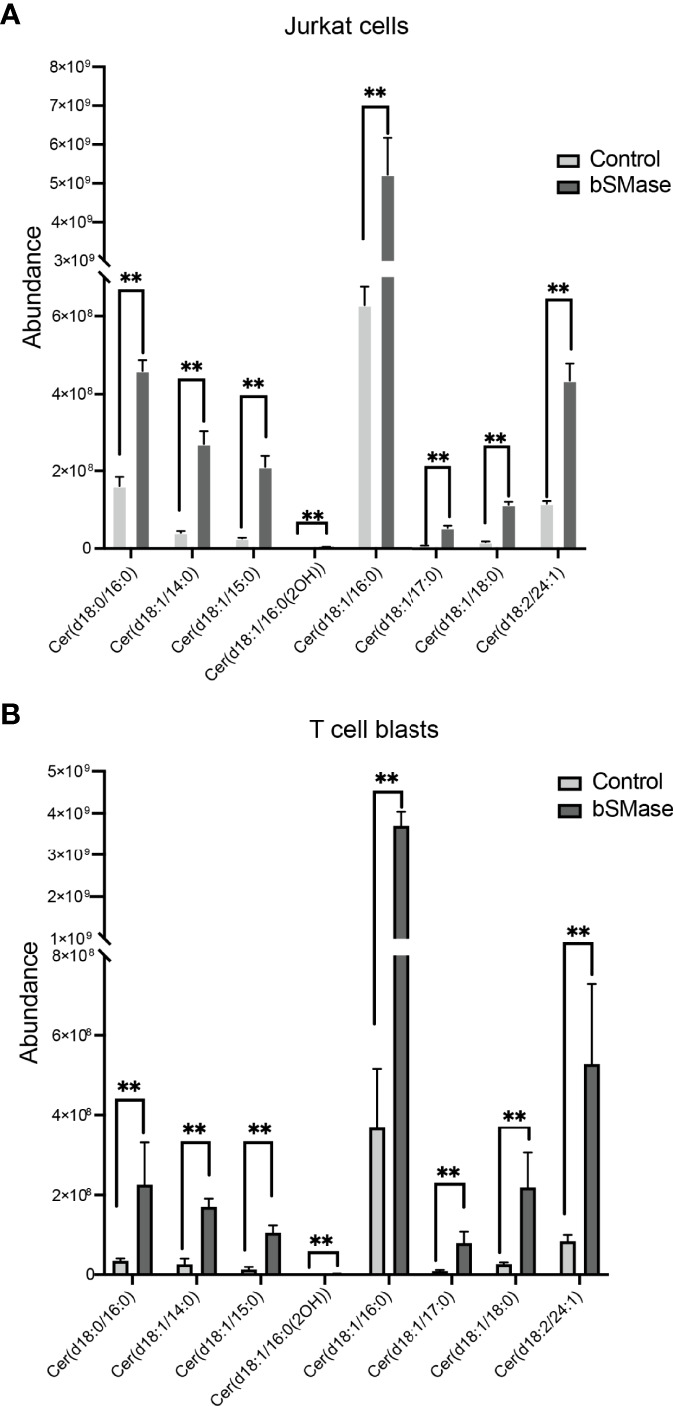
bSMase treatment of Jurkat cells and T cell blasts triggers a profound accumulation of ceramides. Accumulation of multiple ceramide species (Fold Changes > 6 in one of the comparisons) in bSMase-treated samples when compared with the control group for Jurkat cell lines **(A)** and T cell blasts **(B)**. The error bars represent the standard error of the mean (SEM). n = 5 in duplicates, **p ≤ 0.01.

To investigate how sphingomyelin ablation affects CXCR4 dynamics at the cell membrane, we treated Jurkat cells and primary T cell blasts for prolonged periods with bSMase (0.5 U/ml, 3 hours, 37°C, 5% CO_2_), and examined its effect on cell migration using Boyden chambers. Results showed a significant reduction of CXCL12-triggered migration in both cell types after bSMase treatment (**Figures 1A, B**). bSMase treatment was not toxic to cells, as evaluated by propidium iodide and annexin-V/7AAD staining and flow cytometry (**Supplemental Figure 2**). Lipidomic analysis of cells using UHPLC-ESI-QTOF MS and MS/MS, in both positive and negative ion mode, demonstrated the complete breakdown of sphingomyelins under these experimental conditions, and the corresponding accumulation of derived ceramides: 18:0/15:0, 18:1/16:0, 18:1/17:0 and 18:2/24:1 (**Figures 2A, B**; **Supplemental Figures 3A, B**). These results indicate that prolonged bSMase treatment promotes sphingomyelin depletion in Jurkat cells and T cell blasts with an accumulation of saturated and unsaturated long chain ceramides and suppressed CXCL12-mediated cell chemotaxis.

**Figure 3:**
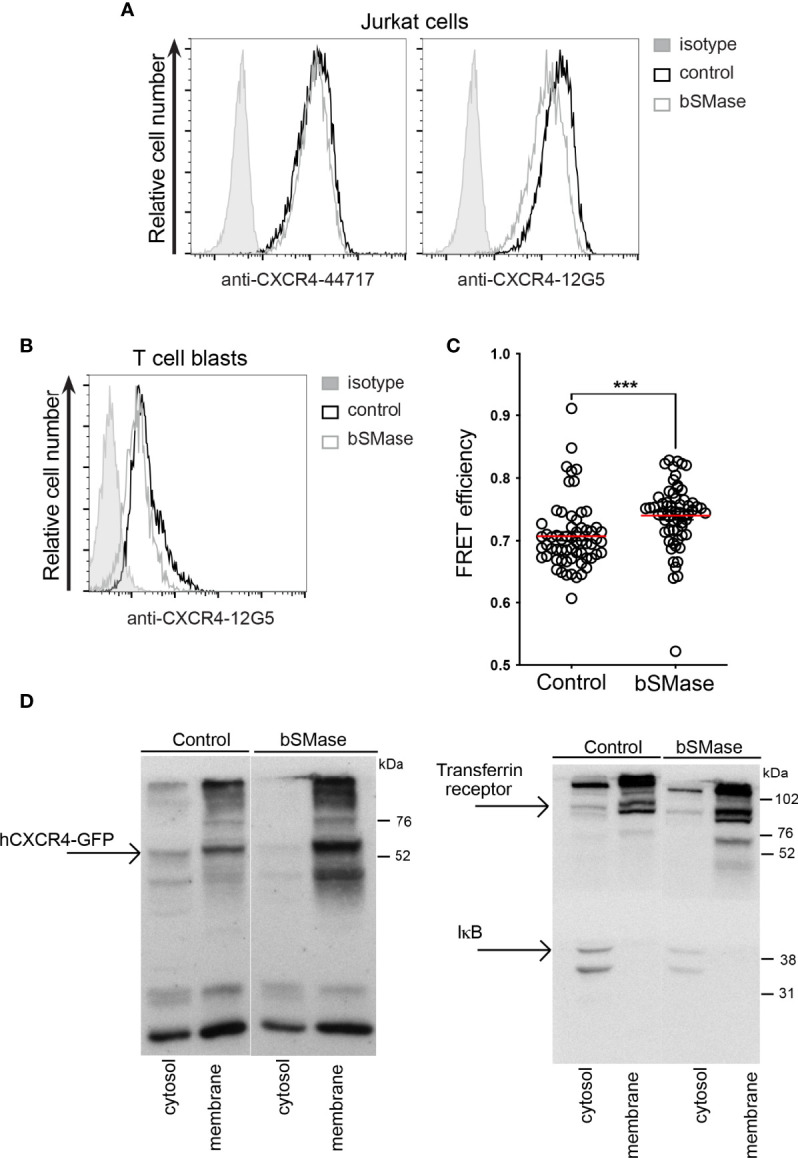
bSMase treatment promotes a conformational change of CXCR4 at the cell membrane. **(A)** Expression of CXCR4 on the surface of untreated or bSMase-treated Jurkat cells, analyzed by flow cytometry using specific antibodies (44717 and 12G5) mAbs. Data show a representative experiment of 6 performed. **(B)** Expression of CXCR4 on the surface of untreated or bSMase-treated T cell blasts, analyzed by flow cytometry using 12G5 mAb. Data show a representative experiment of 6 performed. **(C)** HEK-293T cells transiently transfected at a fixed 9:1 CXCR4-YFP : CXCR4-CFP ratio were treated with bSMase or its solvent (50 mM Tris-HCl; pH 7.5) as a control, and FRET efficiency was evaluated (n = 3, mean ± SEM, ∗∗∗p ≤ 0.001). **(D)** CXCR4-AcGFP-transfected Jurkat-/- cells were untreated or treated with bSMase before fractionation into cytosolic and membrane fractions for western blot analysis with anti-GFP mAb (left) and anti-IKB and anti-transferrin receptor antibodies (right).

Cholesterol interacts with the saturated acyl chains of sphingolipids and phospholipids, conferring specific biophysical properties that increase cohesion and packing of neighboring lipids and proteins. We determined whether the accumulation of ceramides affected the conformation of CXCR4 at the cell membrane. Untreated and bSMase-treated JK cells and T cell blasts were stained with two distinct anti-CXCR4 conformational mAbs (12G5 and 44717 mAbs) (39) and their binding was analyzed by flow cytometry. Results indicated that bSMase treatment reduced 12G5 mAb binding, whereas the binding of 44717 mAb remained unaltered (**Figures 3A, B**). These data suggest that bSMase treatment did not alter CXCR4 levels at the cell membrane but promoted a conformational change on the receptor affecting the epitope recognized by 12G5 mAb. In addition, we performed FRET experiments on untreated or bSMase-treated (0.5 U/ml, 3 hours, 37°C, 5% CO_2_), HEK-293T cells transiently cotransfected with CXCR4-CFP and CXCR4-YFP. Results showed that FRET efficiency was higher in cells treated with bSMase than in untreated cells (**Figure 3C**), confirming the notion that aberrant ceramide accumulation at the cell membrane modifies the conformation of CXCR4 dimers that might be due to an increase of the number of CXCR4 complexes at the cell surface, thus corroborating a relationship between CXCR4 and ceramide enriched-platforms.

To further establish that sphingomyelin depletion *per se* does not affect CXCR4 levels at the cell membrane, we examined the subcellular distribution of transfected CXCR4-AcGFP in Jurkat-/- cells untreated or bSMase-treated (0.5 U/ml, 60 minutes, 37°C, 5% CO_2_) prior to lysis. Western blotting of subcellular fractions indicated that the bulk of CXCR4 was present in the membrane fraction in both untreated and bSMase-treated cells (**Figure 3D**, left). Analysis of the distribution of IKB (cytosolic protein) and the transferrin receptor (membrane protein) confirmed no cross-contamination of fractions (**Figure 3D**, right). Overall, the data suggest that ceramide accumulation does not modify the levels of CXCR4 at the cell membrane, but alters its conformation.

### Aberrant Ceramide Accumulation Influences CXCR4 Dynamics

**Figure 4:**
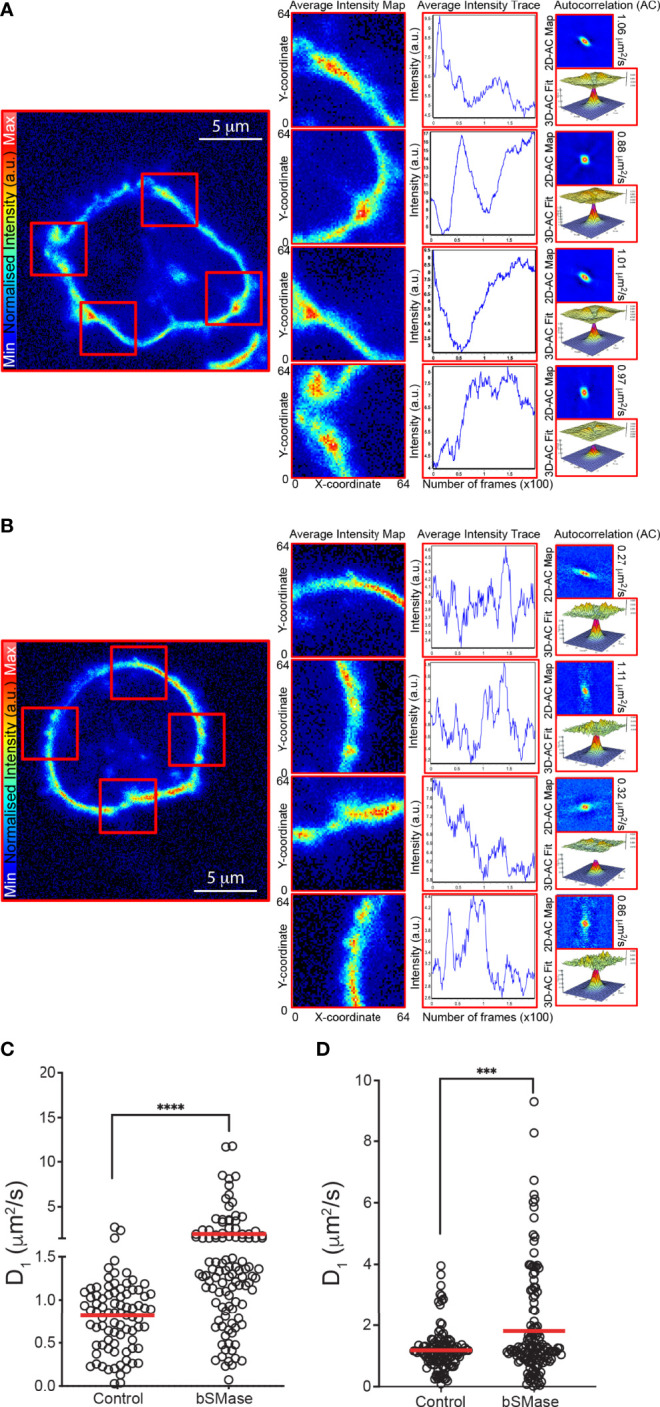
bSMase treatment of Jurkat cells and T cell blasts alters cell membrane fluidity. RICS analysis was performed using the di4-ANEPPDHQ probe at the plasma membrane of untreated and bSMase-treated Jurkat **(A, C)** and T cell blasts (B, D). **(A, B)** Representative average intensity projection maps, average intensity trace, 2D correlation maps, fit to autocorrelation function, ACF, and diffusion coefficient from RICS analysis are shown for each cell type. Scale bar, 5 μm. **(C, D)** Diffusion values obtained by RICS from untreated and bSMase-treated cells, mean is indicated (red) (n = 3, with at least 10 cells analyzed per experiment and condition; n.s., not significant, **p ≤ 0.01, ***p ≤ 0.001, **** p ≤ 0.0001).

**Figure 5:**
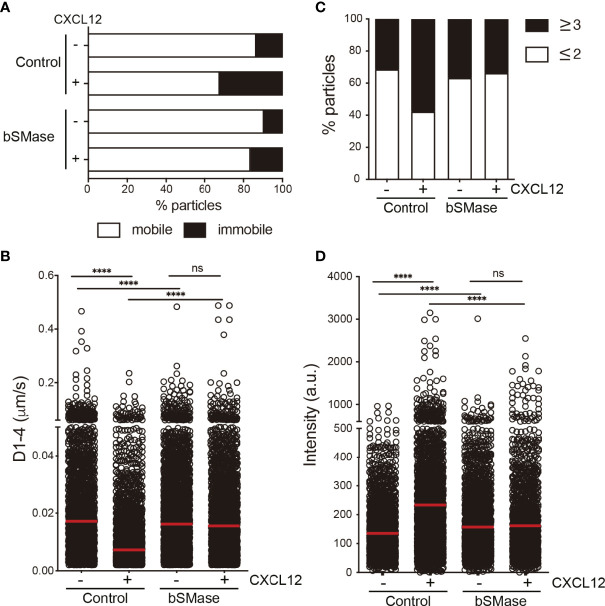
CXCL12 has no effect on CXCR4 dynamics or nanoclustering in bSMase-treated cells. **(A–D)** SPT analysis of CXCR4-AcGFP in untreated or bSMase treated JK^-/-^X4 cells on fibronectin (FN) or FN+CXCL12-coated coverslips (2,064 particles in 79 cells on FN; 2,199 in 60 cells on FN+CXCL12 in untreated JK^-/-^X4 cells; 2,750 in 103 cells on FN; 2,122 in 88 cells on FN+CXCL12 in bSMase-treated JK^-/-^X4 cells; n = 2). **(A)** Percentage of mobile and immobile CXCR4-AcGFP particles at the cell membrane. **(B)** Diffusion coefficients (D_1–4_) of mobile single trajectories, with median (red line) corresponding to untreated or bSMase-treated JK^-/-^X4 cells as in **(A)** (n.s., not significant, ****p ≤ 0.0001). **(C)** Frequency of CXCR4-AcGFP particles expressing monomers plus dimers (≤ 2) or nanoclusters (≥ 3), expressed as a percentage and calculated from mean spot intensity (MSI) values of each particle in cells as in **(A)**, as compared with the MSI value of monomeric CD86-AcGFP. **(D)** Intensity distribution (arbitrary units [a.u.]) from individual CXCR4-AcGFP trajectories on cells as in **(A)**. Mean is indicated (red) (n = 2; n.s., not significant, ****p ≤ 0.0001).

Ceramides influence membrane fluidity and regulate cell migration (40, 41). The effects of ceramide on membrane fluidity are, nonetheless, complex and depend on several factors such as the acyl chain length (42), saturation (43), and the ratio of long chain and very-long chain species present in the cell membrane (44). We thus used RICS with the Di-4-ANEPPDHQ lipid probe to evaluate membrane fluidity by means of membrane diffusion. This lipophilic dye is also a reporter for lipid lateral packing (33, 34). Jurkat cells (**Figures 4A, C**) and T cell blasts (**Figures 4B, D**) untreated or treated with bSMase (0.5 U/ml, 3 hours, 37°C, 5% CO_2_) were labeled with Di-4-ANEPPDHQ and RICS was performed after confocal microscopy. Results indicated that membrane diffusion was higher in cells treated with bSMase than in untreated controls (**Figure 4**), suggesting a more fluid environment. The increase in unsaturated ceramides (C18:1 and C18:2) detected in the cells as a consequence of bSMase treatment (**Figure 2A**) might explain the evident higher membrane diffusion (43). To analyze the influence of the aberrant ceramide accumulation on CXCR4 clustering and lateral diffusion, we next generated SPT trajectories of CXCR4-AcGFP using TIRF microscopy in transiently-transfected Jurkat^-/-^ cells (JK^-/-^X4) (29), untreated or treated with bSMase. We first determined appropriate expression conditions for detecting and tracking individual CXCR4 spots, as described (8). Analysis of receptor trajectories (37), indicated that bSMase treatment had no effect on CXCR4 dynamics under steady-state conditions. A high proportion of CXCR4 particles were mobile (~82% in untreated cells *vs* ~85% in bSMase-treated cells) in both bSMase-treated and untreated JK^-/-^X4 cells (**Figure 5A**). The median value of the short time-lag diffusion coefficient (D_1-4_) for CXCR4 trajectories was 0.017 μm^2^/s in untreated cells and was slightly slower (0.016 μm^2^/s) in bSMase-treated cells (**Figure 5B**). However, whereas CXCL12 promoted a significant reduction in overall receptor diffusivity in control cells (CXCL12, median D_1-4_ = 0.007 μm^2^ s^–1^) and increased the percentage of immobile particles from ~17% (basal) to ~30% (CXCL12), it had no effect on bSMase-treated cells (CXCL12, median D_1-4_ = 0.016 μm^2^ s^-1^), with ~15% of immobile particles (basal) and ~18% after CXCL12 stimulation (**Figure 5A**). To determine the receptor number in individual trajectories, we measured the average fluorescence intensity for the first 20 frames of each trajectory and used the intensity of the monomeric protein CD86-AcGFP as reference (45) (**Supplemental Figures 1A,B**). In steady-state, we found predominantly monomers and dimers in both type of cells (~65% for control *vs* ~62% for bSMase-treated cells), with a similar percentage of complexes of ≥3 receptors (~35% *vs* ~37%, respectively) (**Figures 5C, D**). Accordingly, basal MSI for CXCR4 was comparable in both cases (135.18 a.u. in control *vs* 156.99 a.u. in bSMase-treated cells). As previously shown (8), CXCL12 activation promotes CXCR4 membrane nanoclustering in control cells (~60% of nanoclusters of ≥3 receptors), but it failed to do so in bSMase-treated cells (~37%) (**Figures 5C, D**). These findings indicate that the lipid equilibrium at the cell membrane is essential for the spatiotemporal regulation of CXCR4 nanoclustering and dynamics.

### Prolonged Neutral Sphingomyelinase Treatment Alters T Cell Directed Migration

**Figure 6:**
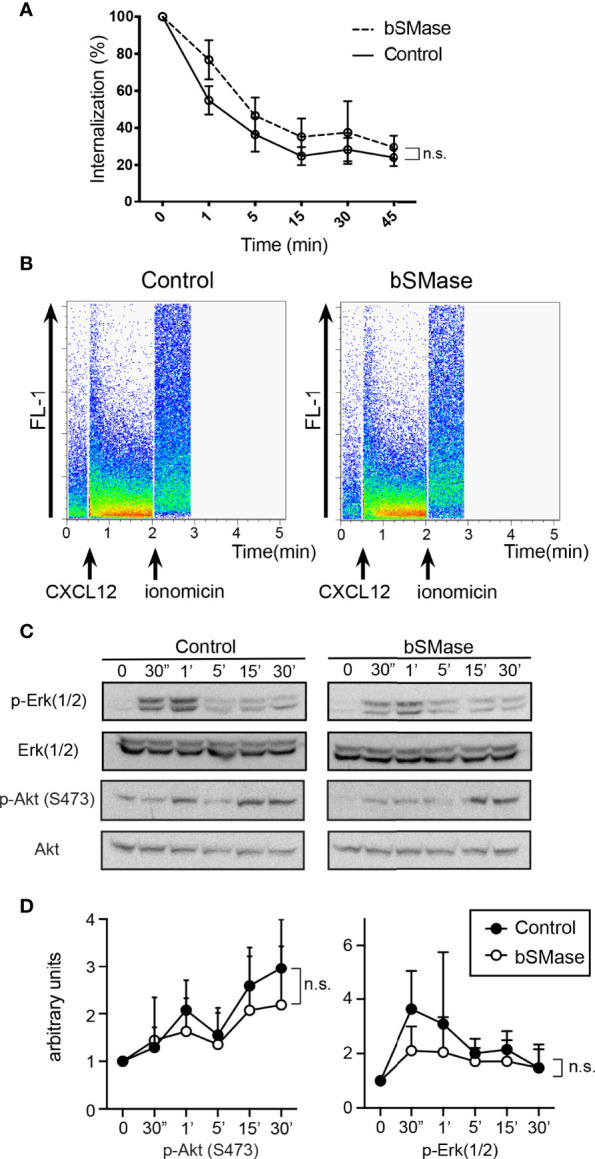
bSMase treatment has no effect on CXCR4 internalization, CXCL12-mediated Ca^2+^ flux and PI3K or ERK1/2 activation in Jurkat cells. **(A)** Cell surface expression of CXCR4 in untreated and bSMase-treated Jurkat cells after stimulation with CXCL12 (50 nM) at different time points, analyzed by flow cytometry using the 44717 anti-CXCR4 antibody. Results show mean ± SEM of the percentage of CXCR4 expression at the cell surface (n = 3, n.s., not significant). **(B)** CXCL12-mediated Ca^2+^ flux in untreated or bSMase-treated Jurkat cells. A representative flow cytometry plot of each condition is shown (n = 3). **(C)** Western blot analysis of ERK1/2 and Akt phosphorylation in untreated and bSMase-treated Jurkat cells stimulated with CXCL12 (50 nM) at the indicated time points (n = 3). **(D)** Densitometric evaluation of western blot experiments (n=3), performed with ImageJ software.

Because ceramide-rich platforms are implicated in a variety of signaling cascades in immune cells, including those related to chemokine receptors (46), we next evaluated whether the effect of bSMase treatment on CXCR4 nanoclustering and dynamics influences CXCR4-mediated functions. Possible differences in CXCR4 internalization due to bSMase treatment were discarded by flow cytometry analysis of Jurkat cells stained with an anti-CXCR4 monoclonal antibody (**Figure 6A**), which showed that the receptor could still bind CXCL12 and activate some cell functions independently of the effects of ceramide accumulation on CXCR4 dynamics. We also observed that CXCL12-triggered Ca^2+^ flux was similar in both untreated and bSMase-treated Jurkat cells (**Figure 6B**) as was CXCL12-mediated Akt phosphorylation (**Figures 6C, D**). We also detected that ERK1/2 phosphorylation, although slightly reduced was not completely impaired by bSMase treatment (**Figures 6C, D**). Altogether these data indicate that sphingomyelin depletion blocked some signaling pathways whereas other were still active.

**Figure 7:**
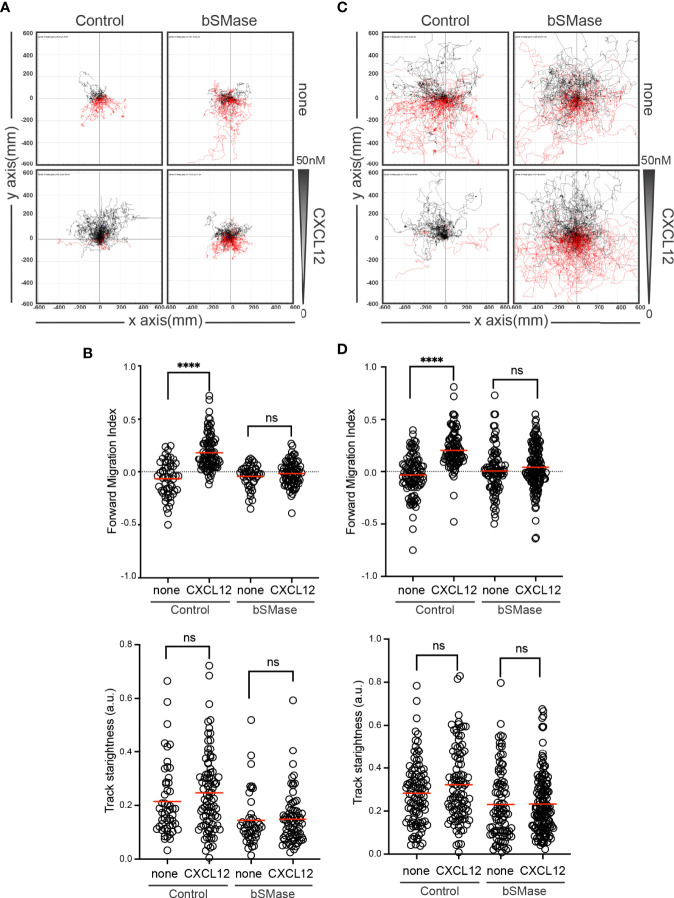
CXCL12 fails to trigger directed migration of bSMase-treated cells. **(A, B)** Migration of untreated or bSMase-treated Jurkat cells on fibronectin-coated μ-chambers in response to a CXCL12 gradient (n = 2, in duplicate, with at least 50 cells tracked in each condition). **(A)** Representative spider plots showing the trajectories of tracked cells migrating along the gradient (black) or moving in the opposite direction (red). Black and red dots in the plots represent the final position of each single tracked cell. **(B)** Quantification of the forward migration index and the Track Straightness of experiments performed in **(A)**. Figures show the data of individual cells, with the mean indicated (red) (n.s., not significant; ∗∗∗∗p ≤ 0.0001). **(C, D)** Migration of untreated or bSMase-treated T cell blasts on fibronectin-coated μ-chambers in response to a CXCL12 gradient (n = 3, in duplicate, with at least 50 cells tracked in each condition). **(C)** Representative spider plots showing the trajectories of tracked cells as in **(A)**. **(D)** Quantitative evaluation of the forward migration index and the Track Straightness of experiments performed in **(C)**. Figures in **(D)** show the data of individual cells, with the mean indicated (red) (n.s., not significant; ∗∗∗∗p ≤ 0.0001).

Because the absence of CXCR4 nanoclustering has been related to defective directed cell migration (29), we analyzed ligand-mediated directed cell migration on fibronectin-coated μ-chemotaxis chambers. Results showed that whereas untreated Jurkat cells sensed the CXCL12 gradient, bSMase-treated Jurkat cells did not (**Figure 7A**; **Supplemental Videos 1** **,** **2**). Quantification of the results indicated that, compared with untreated Jurkat cells, CXCL12 failed to increase the forward migration index and track straightness in bSMase-treated Jurkat cells (**Figure 7B**). These data further show that while some chemokine-mediated signaling events remain operative in cells with aberrant accumulation of ceramides, directed cell migration does not.

To evaluate whether these changes also occur in primary T cells, we reproduced the prolonged bSMase treatment (0.5 U/ml, 3 hours, 37°C, 5% CO_2_) on T cell blasts. We confirmed the complete breakdown of their sphingomyelins and the accumulation of the corresponding ceramides (**Figure 2B**; **Supplemental Figure 3B**). We also observed a defect in their migratory behavior (**Figure 1B**) and RICS analysis of Di-4-ANEPPDHQ indicated higher membrane diffusion in bSMase-treated T cell blasts than in controls (**Figure 4D**). As we expected, bSMase treatment abrogated the ability of CXCL12 to promote cell polarization in T cell blasts, as shown by ICAM-3 and Phalloidin staining (**Supplemental Figures 4A–C**). The same effect was observed in cells treated with CCL19, indicating that bSMase treatment also diminishes CCR7-mediated cell polarization, as previously shown (26) (**Supplemental Figure 5**). In similar experiments we observed that CXCR4 redistribution to the leading edge in response to CXCL12 or CCL19 was also altered upon pre-treatment with bSMase (**Supplemental Figure 5**). We then determined CXCL12-mediated directed cell migration on fibronectin-coated chemotaxis chambers and observed that whereas untreated T cell blasts sensed a CXCL12 gradient, bSMase-treated cells did not (**Figure 7C**; **Supplemental Video 3** **,** **4**). Under these conditions, CXCL12 failed to increase the forward migration index and track straightness (**Figure 7D**). Overall, these data indicate that the aberrant accumulation of ceramides also abrogate the ability of primary T cell blasts to sense CXCL12 gradients.

## Discussion

Cell migration plays a critical role in multiple biological processes, including development, angiogenesis, immune response, wound healing and cancer metastasis, and cells are often directed to migrate towards targets by sensing gradients in the concentration of chemoattractants. Directed cell migration integrates receptors, signal transduction pathways and cytoskeleton dynamics elicited by directional cues, and these events are affected by environmental inputs. The process requires cell polarization and lamella formation in the direction of migration (47), chemokine receptors redistribution to sense the gradients, clustering of integrins to form adhesions that connect the extracellular matrix to the actin cytoskeleton, and tension in the cytoskeleton generated by myosin II (48).

Chemokine receptors are members of the GPCR superfamily (49), and their ligands orchestrate cell migration, cell guidance and chemoattractant gradient formation. In migrating cells, chemokine receptors polarize to the leading edge (50) where they act as sensors for directed migration. The spatial organization of chemokine receptors include monomers, dimers and higher-order oligomers, and these dynamic structures define chemokine-mediated cell responses (8, 51). Chemokine receptors are integral membrane proteins and, accordingly, their interactions with membrane lipids are important determinants in their structure and function.

Changes in cholesterol levels affect the conformation and lateral mobility of both GPCRs within the lipid bilayer and membrane-associated signaling G proteins (52, 53). Cholesterol depletion from membranes attenuates chemokine binding and abrogates CCR5 signaling (54, 55), whereas inclusion of cholesterol increases CXCL12 binding to solubilized CXCR4 (56, 57). Sphingomyelin levels in lipid rafts also regulate CXCL12-induced cell migration (46). Sphingomyelin hydrolysis by neutral sphingomyelinase leads to the formation of ceramide, which in turn promotes cell polarization and motility (58). Local ceramide-enriched membrane microdomains regulate T-cell homeostatic activity and, upon stimulation, compartmentalize receptors, membrane proximal signaling complexes and cytoskeletal dynamics, which are all essential processes for initiating T-cell motility (16). Nonetheless, overactivation of sphingomyelinases causes ceramide mislocalization, loss of adhesion, and cytoskeletal paralysis (59), treatment with exogenous nanoliposomal short-chain ceramides has been shown to inhibit breast cancer cell migration (60) and the use of a potent glucosylceramide synthase inhibitor prevents CXCR4-dependent cell migration (61). A previous report (26) showed that NSM ablation interferes with T cell polarization both at an overall morphological level and through redistribution of CXCR4, we now detected a similar cell phenotype by triggering aberrant ceramides accumulation and altered dynamics of this chemokine receptor at the cell membrane that abrogated the ability of the cells to sense the chemoattractant gradient. These contrasting results suggest that the correct equilibrium between sphingomyelins and ceramides at the cell membrane is essential to maintain fully functional receptors. Specific changes in the lipid composition restricted to local microdomains and activated by receptors in these microdomains are essential for optimal cell functions. Although the direct activation of sphingomyelinases by chemokine receptors has not been yet reported, ligation of CD3 and CD28 is known to activate NSM2 and acid sphingomyelinase, ASM (62, 63) and both have been shown to play an important role in modulating T-cell biology (16).

Here, we evaluated the triggering effects of sphingomyelins and ceramides on CXCR4 conformation and dynamics at the cell membrane of T cells. Lipidomics analysis demonstrated that prolonged treatment with bSMase promotes a profound depletion of sphingomyelins in T cells, and increases the levels of the corresponding ceramides, particularly those of long monounsaturated chains. FRET studies in steady-state cells clearly showed that sphingomyelin ablation increases FRET efficiency between CXCR4 pairs, suggesting a conformational change in the receptors maybe related to increased number of CXCR4 complexes, that was confirmed by a reduction of 12G5 mAb binding in bSMase-treated cells whereas 44717 mAb binding remained unaltered. A previous study in mouse embryonic fibroblasts found that deficiency of sphingomyelin synthase, the enzyme involved in sphingomyelin biosynthesis, potentiates CXCR4 dimerization and influences CXCL12-mediated functions (46). Subcellular fractionation studies also confirmed that CXCR4 levels at the cell membrane fraction was invariable after bSMase treatment.

CXCR4 is embedded in the lipid membrane and, therefore, its nanoclustering and dynamics might be influenced by membrane fluidity; specifically, by the fatty acyl groups of the amphipathic lipids and their degree of saturation (64). Studies on GPCRs have revealed that membrane phospholipids can modulate receptor oligomerization (65) and allosteric activation (66). Along this line, we previously showed that CXCL12-mediated CXCR4 nanoclustering is essential for achieving complete receptor functions (8). Although receptor monomers and dimers can still trigger some signaling pathways (Ca^2+^ flux or ERK1/2 and PI3K activation), receptor nanoclustering is required for the correct orientation of cells towards chemokine gradients. We found in the present study that bSMase treatment altered CXCR4 dynamics and its ligand-mediated nanoclustering in T cells. Indeed, CXCL12 failed to trigger CXCR4 nanoclustering, and also failed to increase the number of immobile particles and reduce its diffusion coefficient. We also found a significant slightly slower diffusion coefficient in steady-state bSMase treated cells compared to the untreated cells, but more experiments are needed to determine whether these minimal differences have some biological relevance. These are essential processes for CXCL12-mediated cell functions. We previously observed that CXCL12-mediated CXCR4 nanoclustering is required to support ligand-mediated direct cell migration (8, 29). Our data thus suggest an essential role for the lipid composition in the local organization and function of CXCR4. Several reports have demonstrated the presence of chemokine receptors in lipid-enriched membrane domains and their role in chemokine function. For example, HIV-1 gp120 interacts and clusters with CD4 and CCR5 or CXCR4 in cholesterol-rich microdomains within the plasma membrane to initiate viral entry (67). Our data also indicate that sphingomyelin ablation abolishes CXCL12-mediated direct migration in T cells, an effect controlled by ligand-mediated receptor nanoclustering. The exposure of dendritic cells to inflammatory signals, reduces their cholesterol levels, enhances the presence of CCR7 oligomers on the cell surface, and promotes efficient cell migration, an effect that is reproduced by statins, which moderately reduce cholesterol levels (68). However, it should be considered that a dramatic reduction of cholesterol levels can also affect the conformational integrity of the chemokine receptors (54).

We also observed that prolonged bSMase treatment affected T-cell polarization, suggesting defects in the connectivity between CXCR4 and the actin cytoskeleton. It is well known that lipid-enriched domains at the cell membrane are platforms for signal initiation and are essential in processes involved in actin cytoskeleton modulation (23). Furthermore, we reported that a correct equilibrium in the actin cytoskeleton dynamics results critical for CXCL12-mediated CXCR4 nanoclustering. Cell treatment with latrunculin A, a drug that blocks F-actin polymerization, abrogates CXCL12-mediated CXCR4-nanoclustering and lateral mobility (8).

In summary, the correct equilibrium of lipid levels within the plasma membrane is essential for membrane fluidity and proper chemokine receptor function. Our results indicate that targeting receptor nanoclustering and lipids could interfere with chemokine functions in a number of pharmacologically relevant situations, such as those including cell recruitment to invade tissues in autoimmune processes or cancer metastasis.

## Data Availability Statement

The datasets presented in this study can be found in online repositories. The names of the repository/repositories and accession number(s) can be found below: Metabolomics Workbench platform (access no. ST002150; https://www.metabolomicsworkbench.org).

## Author Contributions

Author contributions: SG, EG-C, JMR-F, and MM designed all aspects of the study; SG, EG-C, GD’A, AQ-F, and PL performed experiments; BSP performed FRET experiments; EG-C and JB performed and analyzed membrane fluidity experiments; CG-R and CB performed lipidomic analysis; SG, EG-C, JMR-F, and MM wrote the manuscript. All authors contributed to the article and approved the submitted version.

## Funding

This work was supported by grants from the Spanish Ministry of Science and Innovation (PID2020-114980RB-I00). SG and AQ-F are included in the doctoral program of the Department of Molecular Biosciences, Universidad Autónoma de Madrid, E-28049, Madrid, Spain. SG and AQ-F are supported by grants of the Spanish Ministry of Science and Innovation (FPI program, PRE2018-083201 and PRE2019-087966, respectively). EG-C was supported by the program Apoyos Centros de Excelencia S.O. of the Spanish Ministry of Science and Innovation (SEV-2017-0712). We also wish to acknowledge the technical help of the Advance Light Microscopy Unit at the CNB/CSIC.

## Conflict of Interest

The authors declare that the research was conducted in the absence of any commercial or financial relationships that could be construed as a potential conflict of interest.

## Publisher’s Note

All claims expressed in this article are solely those of the authors and do not necessarily represent those of their affiliated organizations, or those of the publisher, the editors and the reviewers. Any product that may be evaluated in this article, or claim that may be made by its manufacturer, is not guaranteed or endorsed by the publisher.
