# Supplementary material for: Sphingomyelin Depletion Inhibits CXCR4 Dynamics and CXCL12-Mediated Directed Cell Migration in Human T Cells
Source: Front Immunol. 2022 Jul 12;13:925559. doi: 10.3389/fimmu.2022.925559 (PMC9315926; doi:10.3389/fimmu.2022.925559)
Supplement: Supplementary file 1 [file DataSheet_1.pdf]

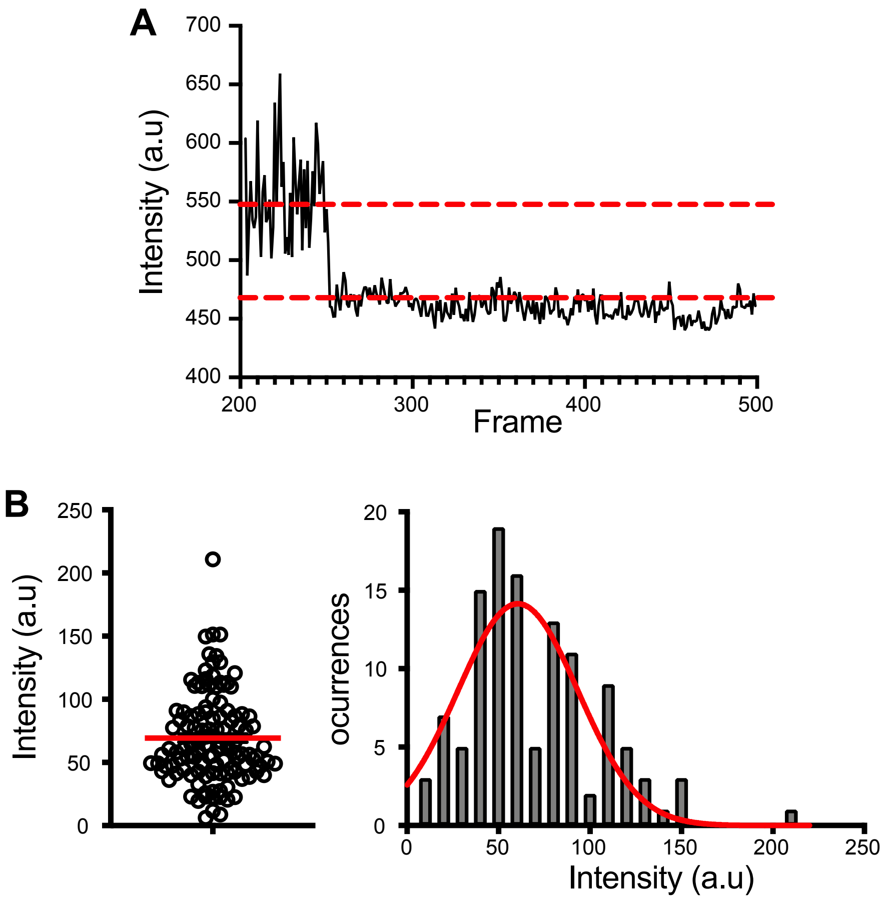

Supplementary Figure 1. Characterization and calculation of reference parameters for particles intensity, related to Figure 5. (**A**). Representative one-step photobleaching of the monomeric CD86-AcGFP particle. (**B**) Fluorescence intensity histogram from monomeric CD86-AcGFP single particles, detected by one photobleaching step evaluation (data from 118 trajectories in 21 cells in 3 separate experiments). The reference fluorescence intensity value for monomer (69.33 ± 3.26 a.u.) was obtained from the Gaussian fit (red line) of the histogram.

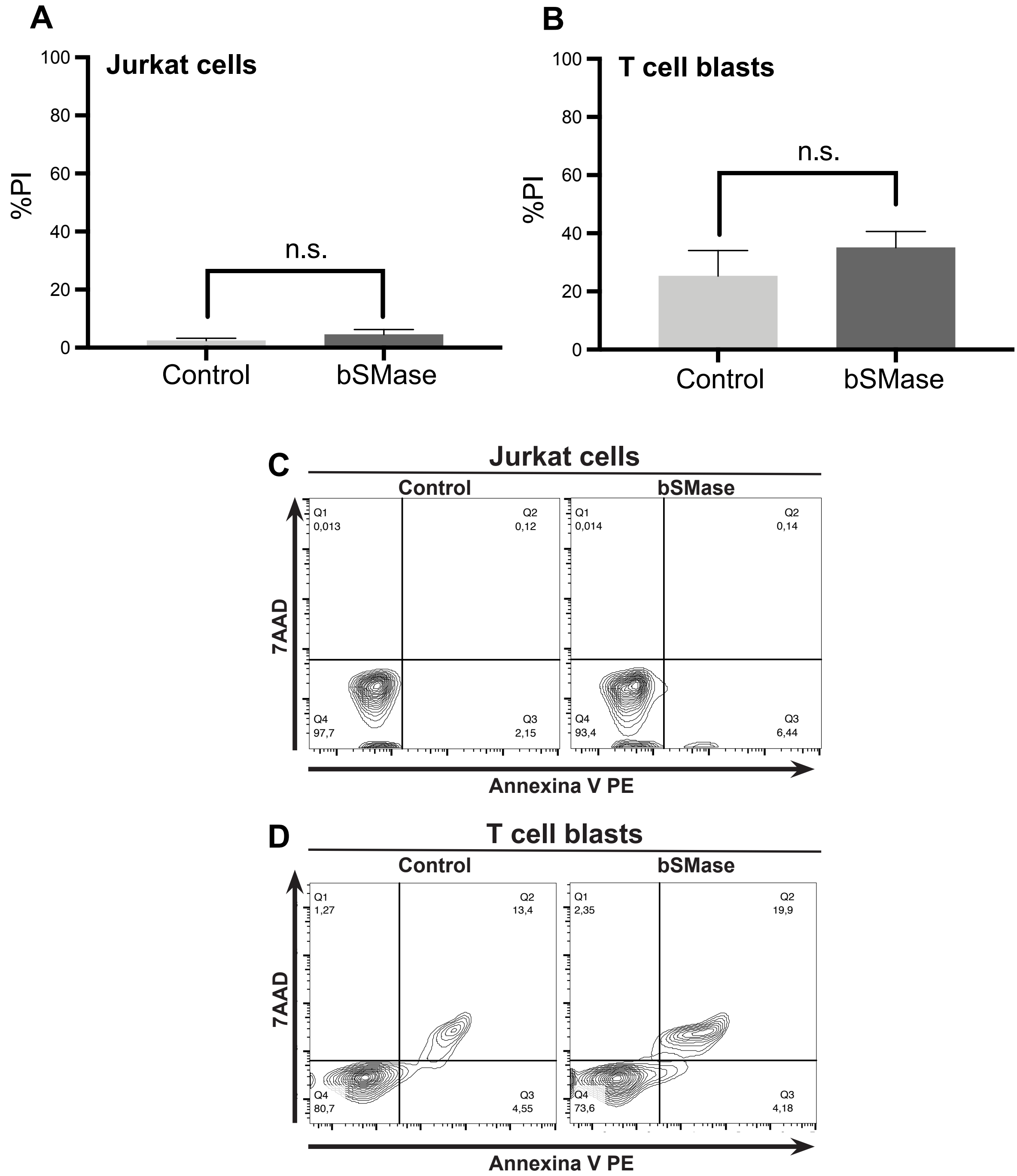

Supplementary Figure 2. bSMase treatment has no significant effect on cell viability. Viability of Jurkat cells (**A, C**) and T cell blasts (**B, D**) exposed to bSMase (0.5 UI/ml, 3 hours, 37ºC) and determined by propidium iodide (**A, B**) or Annexin-V/7AAD (**C, D**) incorporation and flow cytometry. (**A, C**) Data represent the mean ± SEM of 3 independent experiments carried out in triplicate (n.s., not significant). (**B, D**) Show a representative contour plot of 3 independent experiments perfomed.

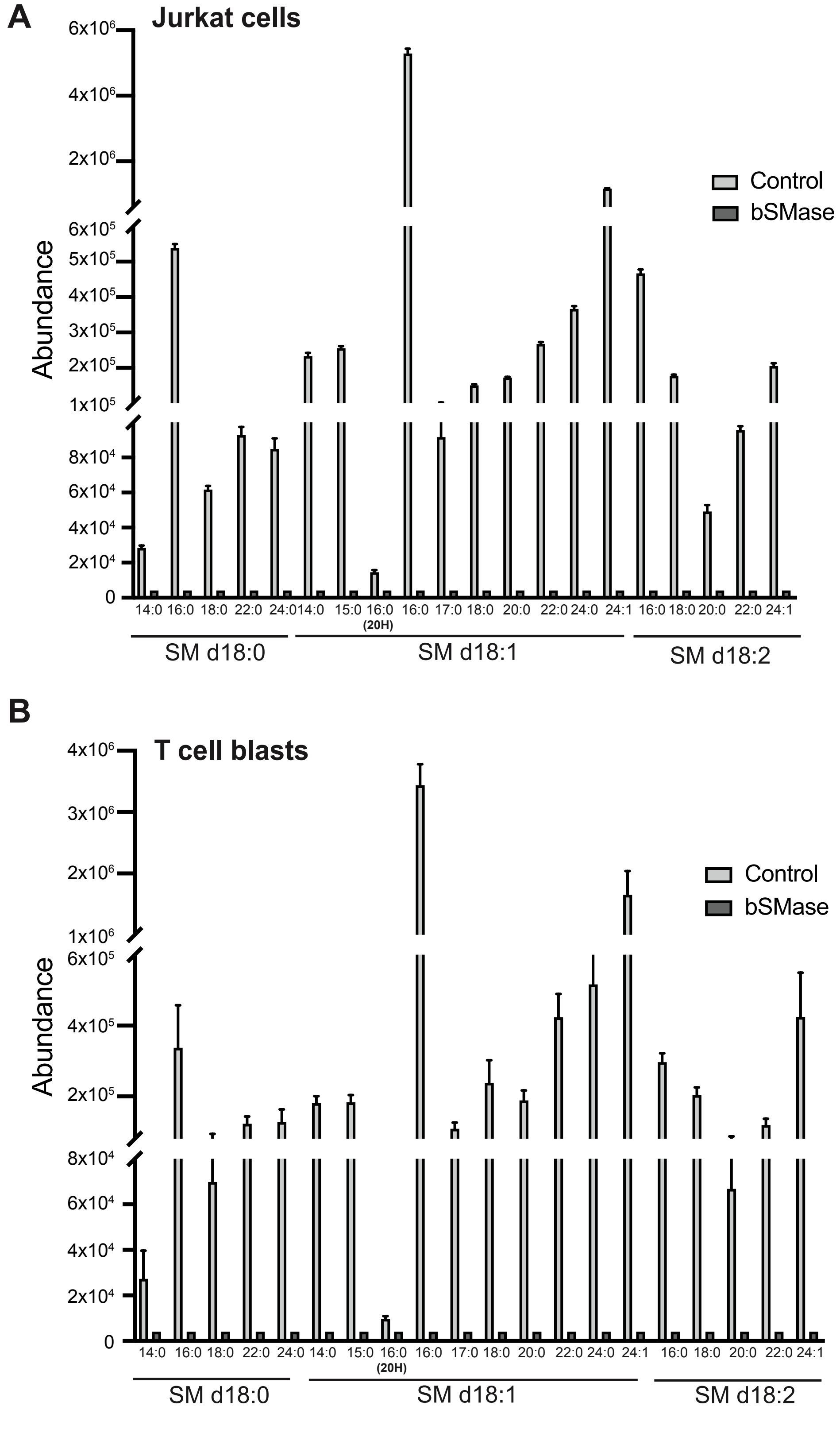

Supplementary Figure 3. Lipidomic profile of Jurkat cells and T cell blasts. Profile of sphingomyelins in Jurkat cells (**A**) and T cell blasts (**B**), determined using by UHPLC-ESI-QTOF MS. The error bars represent the standard error of the mean (SEM) from 8 independent experiments.

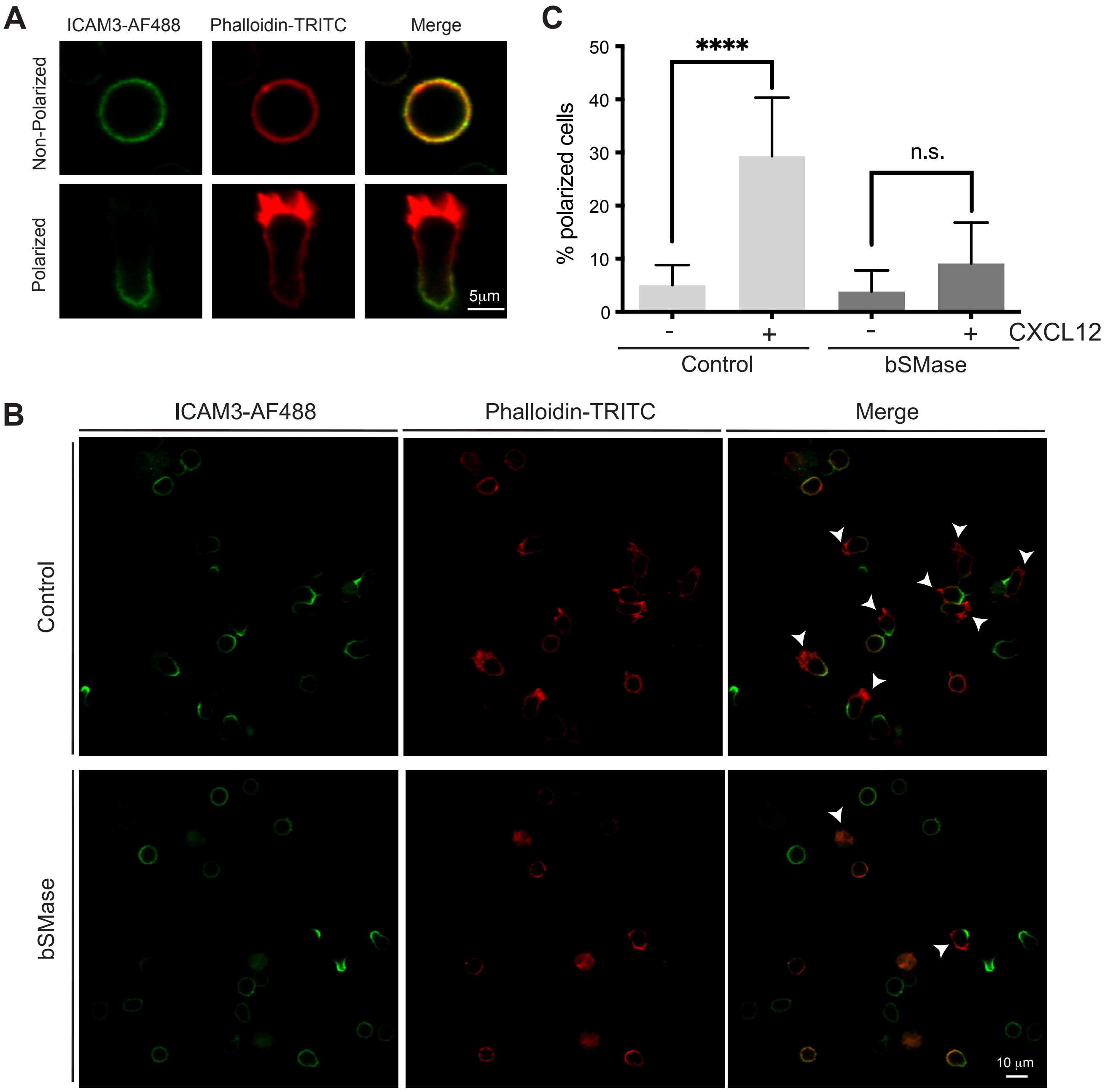

Supplementary Figure 4. bSMase treatment alters polarization of CXCL12-induced T cell blasts. (**A–C**) F-actin (phalloidin-TRITC, red) and anti-ICAM3 (green) visualized by confocal microscopy in T cell blasts untreated or bSMase-treated, adhered to fibronectin and treated or not with CXCL12 (100 nM), as indicated. (n = 2, more than 150 cells analyzed in each condition). (**A**) A representative image of a non-polarized (upper) and a polarized T cell blast (stimulated with CXCL12) showing staining for ICAM-3 (green), Phalloidin (red) and merge panels are shown. Scale bare: 5 µm. (**B**) A representative microscopy image of untreated and bSMase-treated cells activated with CXCL12 is shown. Panels shown correspond to representative ICAM3 (green), Phalloidin (red) and merge images. Scale bare: 10 µm. (**C**) Percentage of polarized T cell blasts is shown. (mean ± SEM, n.s., not significant; ****$p \leq 0.0001$). White arrows indicate phalloidin-enriched lamellipodia.

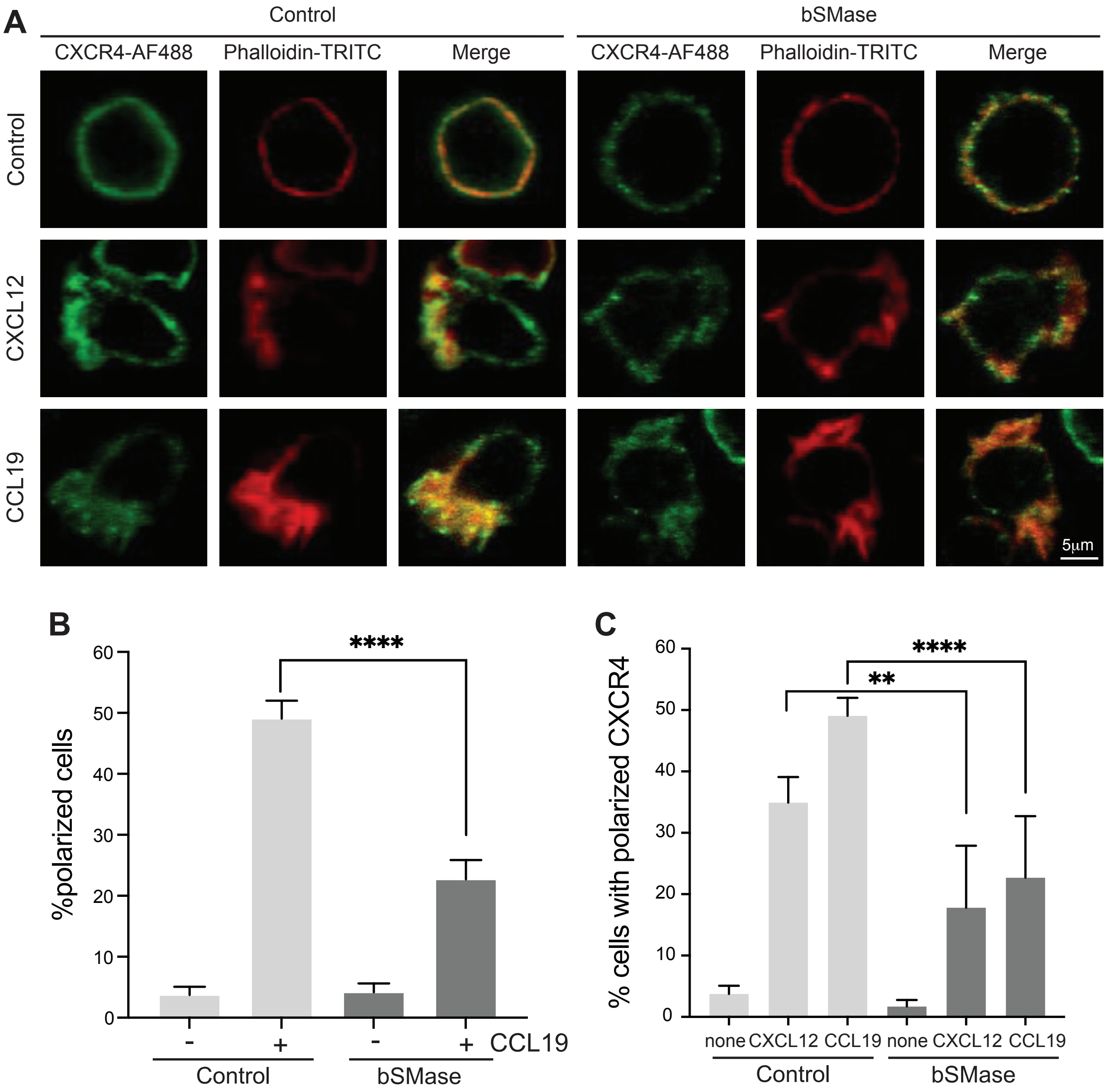

Supplementary Figure 5. bSMase treatment alters polarization of CXCR4 in chemokine-induced T cell blasts. (**A**) F-actin (phalloidin-TRITC, red) and anti-CXCR4 (AF-488, green) visualized by confocal microscopy in T cell blasts untreated or bSMase-treated, adhered to fibronectin and treated or not with CXCL12 or CCL19 (100 nM), as indicated. (n = 2, more than 150 cells analyzed in each condition). Representative images of non-polarized (upper panels) and polarized T cell blasts (stimulated with CXCL12 or CCL19, middle and lower panels) showing staining for CXCR4 (green), Phalloidin (red) and merge panels are shown. Scale bare: 5 μm. (**B**) Percentage of polarized T cell blasts is shown. (mean ± SEM, ****p ≤ 0.0001). (**C**) Percentage of T cell blasts with polarized CXCR4 is shown. (mean ± SEM, ** p≤0.01 ; ****p ≤ 0.0001).

Video S1 (separate file)

Related to Figure 7A. Representative video of untreated JK cell migration on fibronectin-coated μ-Slide Chemotaxis chambers following a CXCL12 gradient on top. Images over time (15 frames/s) are shown. Overlaid trajectories of cells shown in movie were detected and tracked using Fiji software.

Video S2 (separate file)

Related to Figure 7A. Representative video of bSMase-treated JK cell migration on fibronectin-coated μ-Slide Chemotaxis chambers following a CXCL12 gradient on top. Images over time (15 frames/s) are shown. Overlaid trajectories of cells shown in movie were detected and tracked using Fiji software.

Video S3 (separate file)

Related to Figure 7C. Representative video of untreated T cell blasts migration on fibronectin-coated μ-Slide Chemotaxis chambers following a CXCL12 gradient on top. Images over time (15 frames/s) are shown. Overlaid trajectories of cells shown in movie were detected and tracked using Fiji software.

Video S4 (separate file)

Related to Figure 7C. Representative video of bSMase-treated T cell blasts migration on fibronectin-coated μ-Slide Chemotaxis chambers following a CXCL12 gradient on top. Images over time (15 frames/s) are shown. Overlaid trajectories of cells shown in movie were detected and tracked using Fiji software.
